# Unveiling Unexpected Immune Activities Induced by Your Pneumococcal Vaccine

**DOI:** 10.1128/mBio.00137-16

**Published:** 2016-02-23

**Authors:** Julia L. Hurwitz, Elaine Tuomanen

**Affiliations:** Department of Infectious Diseases, St. Jude Children’s Research Hospital, Memphis, Tennessee, USA

## Abstract

In modern-day vaccine design, a good pneumococcal capsular polysaccharide vaccine is measured by its ability to induce opsonic antibodies. These antibodies label bacteria for phagocytosis by neutrophils and thereby overcome the capsule’s barrier function. Doyle and Pirofski have raised a serious challenge to the current paradigm by describing anti-capsular antibodies that are highly protective but nonopsonic [C.R. Doyle and L. Pirofski, mBio 7(1):e02260-15, 2016, doi:10.1128/mBio.02260-15]. In fact, some functions are not related to neutrophils or phagocytosis at all. An increased awareness of these activities is critical not only for accurate comparisons of vaccine candidates but also for improvements in vaccination outcomes in settings of neutropenia. When vaccine developers select a single gatekeeper assay (e.g., an opsonophagocytic assay for bacteria or a neutralization assay for viruses), promising vaccine candidates may be missed. Doyle and Pirofski stress that multiple functions, not just one, should be investigated to enhance discovery of antibody mechanisms and to best assess vaccine-induced correlates of immune protection.

## COMMENTARY

*Streptococcus pneumoniae* is a leading cause of pneumonia, sepsis, and meningitis and remains the most common agent leading to hospitalization in all age groups. The pneumococcus is the major cause of pneumonia, which kills more children than any other illness, accounting for 1 in 5 deaths of children under 5 years of age worldwide. The pneumococcus has also served as an important model organism for understanding the immune response to infection by Gram-positive bacteria.

The major virulence determinant of the pneumococcus is its polysaccharide capsule, which has over 90 antigenically distinct chemical structures or serotypes. One bacterial defense mechanism mediated by the capsule is interference with phagocytosis by neutrophils. Combinations of different purified capsules are the principle components of all licensed pneumococcal vaccines. The vaccines are designed to induce opsonic antibodies that bind capsules and reverse interference by independently recruiting neutrophils (often supported by complement and Fc-Fc receptor [FcR] interactions) to mediate bacterial engulfment and killing. Based on a narrow vision of anti-capsule antibody function, the opsonophagocytic killing assay (OPA or OPKA) is used as a gold-standard measure of vaccine success. In a recent article in mBio (1), Doyle and Pirofski raised a serious challenge to the unifocal concept of what is a “good” vaccine, what is a “good” anti-capsular antibody, and hence, what is a “good” antibody assay.

## CURRENT DOGMA VERSUS NEW VIEW FOR DEFINING A PROTECTIVE ANTIBODY

Doyle and Pirofski describe two mouse monoclonal pneumococcal capsular polysaccharide serotype 3 (PPS3)-specific IgG1 antibodies, 7A9 and 1E2. In previous studies, 7A9 killed pneumococcus in the OPKA (in the presence of mouse neutrophils and complement *in vitro*), while 1E2 did not. Based on the current paradigm that OPKA associates with protection, it was predicted that 7A9 would be better than 1E2 at preventing nasopharyngeal (NP) colonization and bacterial dissemination to the lungs and blood of mice challenged intranasally with a serotype 3 strain of pneumococcus. However, the authors found that when antibodies were delivered passively by the intraperitoneal route, 1E2 significantly reduced NP colonization and prevented early dissemination to lungs and blood, whereas 7A9 did not reduce NP colonization and reduced levels of bacteria in the blood only after 6 days of infection. When delivered intranasally, both antibodies reduced NP CFU, but 7A9 was dependent on the presence of Fc, whereas 1E2 was not. Again, only 1E2 significantly prevented dissemination of bacteria to lungs and blood, a feature that did not depend on Fc.

These data reveal a complexity of antibody function, a complexity that is further indicated by comparing two challenge systems. In a previous publication, 1E2 and 7A9 were tested in a lethal pneumonia model using a different serotype 3 strain, administered intranasally. In that case, both antibodies afforded protection and 7A9 reduced lung and blood CFU better than did 1E2. Protection afforded by 7A9 required FcγRII, whereas protection afforded by 1E2 required a different receptor and was most potent in the presence of macrophages. Clearly, the excellent outcomes mediated by just two monoclonal antibodies were not correlated with the gold-standard assay.

## HOW DOES A NONOPSONIZING ANTICAPSULAR ANTIBODY PROTECT?

There are a number of non-mutually exclusive mechanisms that can explain antibody-mediated protection other than opsonization. Antibodies may agglutinate bacteria *in vivo* (although 1E2 was tested and does not), a feature that may block bacterial transport or support IgG-dependent complement deposition on the bacterial cell surface to subsequently attract neutrophils. Neutrophils may phagocytose bacteria but may alternatively produce extracellular traps (NETs) comprising chromatin and antimicrobial peptides to inhibit bacterial growth (2). The modulation of innate immune responses by antibodies (e.g., the Fc-dependent reductions in interleukin-6 [IL-6] levels associated with 1E2, described by Doyle and Pirofski) also impacts disease. One more possibility is that PPS3-specific antibodies may assist transfer of bacteria to macrophages and macrophage immunomodulation (3). In assays completely unrelated to neutrophils, 1E2 modulated signals associated with quorum sensing and upregulated fratricide in the presence of competence-stimulating peptide (4). Thus, antibodies with nonopsonic activities comprise a potent potential source of protective activity induced by a capsular vaccine. This concept is of great clinical importance in the setting of neutropenia, such as during cancer therapy.

## NOT JUST IgG

An analysis of antibody isotypes other than IgG reveals a further breadth of opsonizing and nonopsonizing functional potentials. Worthy of attention is natural IgM (5), an antibody generally produced by B-1 B cells in mice and by “B-1-like” or “innate-like” B cells in humans (6). It is polyreactive and able to bind common conformational motifs on self- and microbial structures, including multiple different pneumococcal polysaccharide serotypes. It is generally characterized by the presence of an unmutated heavy chain and a long CDR3 and by preferential use of certain V, D, and J family genes. IgM, whether produced by B-1 cells or by the more conventional B-2 B cell subset, mediates a variety of defenses, from the simple aggregation of bacteria and enhancement of complement deposition to the localization of circulating bacteria to splenic marginal zones and modulation of lymphocyte activation/differentiation (7). In mouse models, IgM monoclonal antibodies have been shown to delay the development of invasive pneumococcal disease and to provide protection from lethality (8).

IgA also deserves attention, because this isotype represents the major class of antibodies in mucosal secretions. Like IgM, IgA is produced by B-1 and B-2 B cell lineages and can be polyreactive in specificity. In humans, IgA exists as two subclasses, IgA1 and IgA2, which exist in monomeric and polymeric forms. In the mucosa, IgA is most often dimeric, with two monomers (each comprising two immunoglobulin heavy chains and light chains) stabilized by the J chain. While IgA is relatively weak in terms of complement activation, it has the potential to inhibit bacterial binding to host targets, augment phagocytosis, and modulate other innate and adaptive effector functions. In fact, to facilitate its binding to the host mucosa, *Streptococcus pneumoniae* expresses an IgA1 protease to actively subvert IgA function (9).

Of all the antibody isotypes, IgA is best suited for surveillance of mucosal membranes, because it can traffic from underlying tissue to the lumen through epithelial cells, escorted by the polymeric immunoglobulin receptor. Antibody then bathes the respiratory tract in mucosal secretions and can be tethered to the mucosal surface, stabilized by secretory component (a fragment of the poly-Ig receptor) to act as a first line of defense against bacterial invasion. Clearly, not just the functions but also the locations of antibody functions are diverse.

The activities of antibodies (and of other effectors of the adaptive and innate immune system) combine and synergize to confer an overall protective effect. Just as we use combination drugs to tackle pathogens (and other ailments), so may we use a plethora of immune functions as an armament against bacteria. The OPKA will clearly not reveal these full potentials of the immune system.

## CHALLENGING PARADIGMS

The results of Doyle and Pirofski speak to the extraordinarily broad bioactivities of monoclonal and polyclonal antibodies to one antigen, the capsular polysaccharide, in terms of both quantity and quality of immune functions. The idea that the capsule is involved only in blocking phagocytosis is passé, as is its corollary, that a “good” polysaccharide-based vaccine must be active in an OPKA. Dialogue concerning immune correlates of protection will help both basic researchers and clinicians quantify important metrics for vaccine candidate evaluation. Encouragement by Doyle and Pirofski of dialogue, just as new vaccines containing pneumococcal proteins are positioned in the pipeline to broaden serotype coverage, is timely. The new vaccines will induce antibodies with an expanded array of targets and target functions (e.g., bacterial adherence, invasion, complement binding, matrix interactions, biofilm formation, etc.) and will force further diversification of assays defining a “good” vaccine in efficacy trials. Comprehensive assays may additionally lead to the discovery of new mechanisms and unanticipated cross-involvement of antigens during the infection process (10).

In conclusion, Doyle and Pirofski raise awareness that the use of a single functional assay is generally insufficient to characterize the plethora of immune activities that follow infection or vaccination, even if only with a single antigen. This is especially important when the OPKA is used as the single defining measure, a practice that overlooks non-neutrophil-mediated protective activities that could be critical to survival of immunocompromised patients. The same situation characterizes other vaccine fields, in that selection of a single assay is logistically favorable but does not suffice as a measure of a vaccine’s protective capacity. Individual gatekeeper assays (e.g., assays of phagocytosis for bacteria and of neutralization for viruses), once selected, may hamper the selection of promising vaccine candidates. Instead, sophisticated assays are needed, not only to identify the full functional capacities of vaccine-induced antibodies but also to characterize antibody binding sites and cross-reactive potentials (e.g., to discriminate the serotype-specific from the much-desired, broadly neutralizing antibodies). The work by Doyle and Pirofski prompts us to develop better assays, with which we may develop better vaccines.
